# Problematic mobile phone use, loneliness, and life satisfaction among college students during the COVID-19 lockdown in Shanghai: a cross-sectional mediation analysis

**DOI:** 10.3389/fpubh.2026.1925515

**Published:** 2026-08-18

**Authors:** Qiong Xu, Bin Xu, Junpeng Yan

**Affiliations:** 1 Shanghai Business School, Shanghai, China; 2 Faculty of Health Sciences and Sports, Macao Polytechnic University, Macau, Macao SAR, China; 3 Xianda College of Economics and Humanities, Shanghai International Studies University, Shanghai, China

**Keywords:** college students, COVID-19 lockdown, cross-sectional mediation, life satisfaction, loneliness, problematic mobile phone use

## Abstract

**Introduction:**

During Shanghai's COVID-19 lockdown, college students relied heavily on mobile phones for study, communication, and everyday activities, while normal campus routines and face-to-face contact were sharply restricted. Although problematic mobile phone use, loneliness, and life satisfaction have each received considerable research attention, less is known about how they were related under these unusual conditions. This study therefore examined their cross-sectional associations and evaluated whether loneliness statistically accounted for part of the association between problematic mobile phone use and life satisfaction.

**Methods:**

We conducted a cross-sectional online survey of 1,311 freshmen and sophomores from seven public undergraduate colleges in Shanghai during the 2022 lockdown. PMPU, loneliness, and life satisfaction were assessed using validated scales. Pearson correlations and covariate-adjusted ordinary least squares mediation analyses were performed, with indirect associations estimated using 5,000 percentile bootstrap resamples. Sensitivity analyses examined alternative model specifications.

**Results:**

PMPU was positively correlated with loneliness (*r* = 0.233, *p* < 0.001) and negatively correlated with life satisfaction (*r* = −0.220, *p* < 0.001); loneliness was also negatively correlated with life satisfaction (*r* = −0.476, *p* < 0.001). In the covariate-adjusted model, PMPU was associated with greater loneliness (*a* = 0.205, *p* < 0.001), whereas loneliness was associated with lower life satisfaction (*b* = −0.281, *p* < 0.001). The indirect association was −0.058 [95% bootstrap CI (−0.073, −0.043)], accounting statistically for 45.9% of the total association. Sensitivity analyses yielded similar estimates, although the alternative-direction model also showed a non-zero indirect association.

**Conclusion:**

Greater PMPU was associated with lower life satisfaction among college students surveyed during the Shanghai lockdown, with loneliness statistically accounting for part of this association. However, the cross-sectional design and the non-zero indirect association in the alternative-direction model preclude conclusions about temporal ordering or causal mediation.

## Introduction

The COVID-19 pandemic substantially disrupted university life. During Shanghai's city-wide lockdown in 2022, students had limited face-to-face contact, spent less time outdoors, and completed much of their academic and social activity online. These conditions may have affected student wellbeing, as studies conducted during the pandemic documented anxiety, depression, stress, post-traumatic stress symptoms, and sleep problems among university students across multiple countries (1). Social isolation was also associated with poorer mental health (2), although students' psychological responses varied across different phases of the pandemic (3).

During the pandemic, mobile phones became essential tools for attending classes, obtaining information, maintaining social contact, and organizing daily activities. Young adults are among the most active mobile phone users in China (4), and university students commonly use smartphones for communication, learning, entertainment, and everyday tasks (5). Although these functions are often beneficial, constant accessibility and highly personalized content may make smartphone use difficult to regulate for some students (6).

Several overlapping terms have been used in the literature, including mobile phone dependence, excessive mobile phone use, and problematic mobile phone use (PMPU) (7). In this study, PMPU refers to difficulty controlling mobile phone use, discomfort when access is restricted, and interference with daily responsibilities (8). PMPU is not treated as a formal psychiatric diagnosis. Nevertheless, higher PMPU has been associated with anxiety, depression, sleep problems, emotion-regulation difficulties, reduced productivity, poorer academic performance, and lower wellbeing (9–17). A systematic review and meta-analysis similarly linked problematic smartphone use to adverse mental-health outcomes among children and young people (18), while problematic use was also reported across age groups during the Italian lockdown (19).

Life satisfaction refers to individuals' overall evaluation of their lives according to their own standards and expectations (20). During the pandemic, this evaluation may have been affected by academic disruption, reduced autonomy, uncertainty, and restricted access to campus life. Previous research among Chinese university students found that greater PMPU was associated with lower life satisfaction during the COVID-19 pandemic (21). Loneliness was widely reported during quarantine (22), while pandemic-related disruption was associated with greater distress and lower life satisfaction (23).

Loneliness reflects a perceived gap between desired and actual social relationships rather than simply being alone (24–28). According to Compensatory Internet Use Theory, individuals may turn to the internet or mobile phones when their offline social needs are unmet or when they are trying to cope with distress (29). This perspective may be particularly relevant when face-to-face interaction is restricted and opportunities for peer contact and campus participation are reduced.

Loneliness may encourage individuals to use smartphones or other digital media to compensate for unmet social needs. At the same time, poorly regulated digital use may be associated with reduced offline interaction and a weaker sense of belonging, suggesting that the relationship may operate in both directions (24, 25, 30–36). From a deficient self-regulation perspective, problematic use is characterized not simply by the amount of time spent online, but by difficulty controlling use when it interferes with other goals or responsibilities (37). Studies among university and nursing students have also reported associations of loneliness and social anxiety with problematic mobile phone and social media use (38–40).

Previous studies have generally linked problematic smartphone use with lower life satisfaction and poorer psychological wellbeing. Research on problematic internet and social media use has reported broadly similar patterns, although the strength and form of these associations vary across populations and measures (34–36, 38, 41–50). Studies examining belongingness, fear of missing out, loneliness, and gender-related differences further suggest that relational factors may shape these associations (51, 52). However, much of this evidence is cross-sectional and involves related rather than identical constructs, leaving the direction of the associations uncertain.

Our primary model specified PMPU as the predictor, loneliness as the mediator, and life satisfaction as the outcome. This specification was informed by evidence linking poorly regulated phone use with reduced offline interaction and social disconnection. However, Compensatory Internet Use Theory also supports the reverse ordering, whereby loneliness may promote greater reliance on mobile phones for coping or social compensation. Given the cross-sectional design, we treated the model as a statistical representation rather than a temporal or causal sequence and examined an alternative model with loneliness as the predictor and PMPU as the mediator.

Against this background, we examined the associations among PMPU, loneliness, and life satisfaction in freshmen and sophomores surveyed in Shanghai during the 2022 lockdown. We hypothesized that loneliness would statistically account for part of the association between PMPU and life satisfaction (H1). Specifically, we expected PMPU to be positively associated with loneliness (H2a) and loneliness to be negatively associated with life satisfaction (H2b).

## Methods

### Study design and participants

We conducted a cross-sectional online survey among undergraduate students in Shanghai during the 2022 lockdown. The target population comprised freshmen and sophomores. We excluded records from students outside the eligible grade range, records with unusually short completion times, and records submitted outside the retained data-collection window. The final analytic sample comprised 1,311 students.

### Procedure and ethics

The questionnaire was administered through Wenjuanxing (also known as Questionnaire Star), an online survey platform, and was distributed to students by physical education teachers and college counselors. Submissions were received between May 20 and June 1, 2022. To ensure that the analytic sample reflected the period of strict lockdown and to avoid the transition immediately before restrictions were lifted on June 1, only responses submitted from May 20 to May 30 were retained. All participants provided electronic informed consent before completing the questionnaire. The study was approved by the Ethics Committee of Shanghai University of Sport (approval no. 102772021RT004).

### Measures and scale scoring

PMPU was assessed using Leung's 17-item Mobile Phone Addiction Index (MPAI) (53), which has shown acceptable reliability in Chinese college-student samples (54, 55). The scale covers four dimensions: inability to control craving, anxiety and feeling lost, withdrawal and escape, and productivity loss. Items were rated from 1 (almost never) to 5 (always). Responses were summed to produce a total score ranging from 17 to 85, with higher scores indicating more problematic use. The total score was used in the primary analyses. Cronbach's α was 0.881 in the present sample.

Loneliness was assessed using the 20-item UCLA Loneliness Scale (Version 3) (56), whose factorial structure and longitudinal measurement invariance have been examined in Chinese college students (57). Items were rated from 1 (never) to 4 (often). Positively worded items (1, 5, 6, 9, 10, 15, 16, 19, and 20) were reverse-scored before all item responses were summed. Total scores ranged from 20 to 80, with higher scores indicating greater loneliness. The continuous total score was used in the primary analyses. Cronbach's α was 0.890 in the present sample.

Life satisfaction was measured with the five-item Satisfaction With Life Scale (SWLS), developed by Diener et al. (58). A Chinese study reported acceptable reliability and validity for the SWLS (59), and broader psychometric reviews have also supported its use (60, 61). Each item was rated on a 7-point scale, and the five responses were summed. Scores could range from 5 to 35, with higher values indicating greater life satisfaction. We used the continuous total score in all primary analyses. Cronbach's α was 0.900 in the present sample.

The questionnaire also collected information on gender, age, grade, ethnicity, height, weight, household registration (urban or rural), only-child status, monthly disposable living expenses, relationship status, alcohol use, and tobacco use. Height and weight were recorded in centimeters and kilograms and used to calculate BMI. Relationship status was assessed using a single self-reported item and categorized as neither dating nor married, dating but unmarried, married, or other. Marital status was not independently verified, and no information was collected on relationship quality, duration, cohabitation, or partner support.

### Data cleaning and statistical analysis

Data preparation and analysis were completed in R version 4.6.1 (R Foundation for Statistical Computing, Vienna, Austria) using reproducible scripts. Before analysis, we checked variable types, allowable item ranges, missing values, and the scale scores stored in the dataset. The MPAI, UCLA, and SWLS totals were recalculated from item responses and compared with the stored values. Cronbach's α was the main index of internal consistency, and McDonald's ω_total_ was reported as a supplementary estimate. We summarized continuous variables with means, standard deviations, medians, interquartile ranges, minima, and maxima, and categorical variables with frequencies and percentages. Pearson correlations were reported with two-sided *p* values and 95% confidence intervals; Spearman correlations served as a sensitivity check.

For the main analysis, we fitted ordinary least squares regression models with the MPAI total score as X, the correctly scored continuous UCLA total as M, and the SWLS total as Y. We estimated the a, b, total (c), and direct (c′) paths as unstandardized coefficients. The indirect association was the product of a and b, with uncertainty estimated from 5,000 nonparametric percentile bootstrap samples. Every component equation used the same 1,311 participants and the same covariates: gender, age, grade, ethnicity, BMI, household registration, only-child status, monthly disposable living expenses, relationship status, alcohol use, and tobacco use. Categorical covariates were dummy coded. Monthly living expenses were entered as an ordinal covariate in the primary model. All tests were two-sided, and p < 0.05 was considered statistically significant.

Standardized indirect associations and their percentile bootstrap confidence intervals were calculated on the same scale by recalculating the standardized path coefficients within each bootstrap resample. Confidence intervals were based on the successfully converged bootstrap resamples.

### Sensitivity and diagnostic analyses

We examined several alternative specifications. These included reversing the focal direction so that loneliness was X and PMPU was M, adding fixed effects for the seven participating institutions, dummy coding monthly living expenses, and checking whether numeric parsing of height and weight affected BMI-adjusted estimates. We also created a supplementary binary MPAI screen from items 3, 4, 5, 6, 8, 9, 14, and 15. A response of 3 or higher counted as a positive endorsement, and five or more endorsements were classified as screen-positive (55). This screen was used only for sensitivity analysis and was not treated as a clinical diagnosis. Exploratory analyses considered gender interactions and gender-stratified indirect associations. Model diagnostics included residual plots, the Breusch-Pagan test, adjusted generalized variance inflation factors, Cook's distance, leverage, studentized residuals, and HC3 heteroskedasticity-consistent standard errors.

### Structural equation modeling as a supplementary robustness analysis

We used confirmatory factor analysis (CFA) and structural equation modeling (SEM) as supplementary checks, implemented in lavaan version 0.6–21. PMPU was represented by four MPAI dimension scores, loneliness by four balanced parcels constructed from the correctly scored UCLA items, and life satisfaction by the five SWLS items. We compared a single-factor model, a three-factor model, and a constrained common latent factor model. We then estimated unadjusted and covariate-adjusted latent cross-sectional mediation models with maximum likelihood. Model fit was judged using chi-square, CFI, TLI, RMSEA with its 90% confidence interval, and SRMR. Bootstrap confidence intervals for latent indirect associations used 1,000 samples in the unadjusted model and 500 requested samples in the adjusted model. These models were used to assess robustness, not temporal or causal mediation.

## Results

## Participant flow, participant characteristics, descriptive statistics, and reliability

The raw dataset contained 1,393 questionnaire records. Data screening excluded 64 records from students outside the target grade range, five records flagged for abnormally short completion times, and 13 records submitted outside the retained data-collection window. The final analytic sample included 1,311 freshmen and sophomores.

The final sample comprised 457 men (34.86%) and 854 women (65.14%). The mean age was 19.31 years (*SD* = 0.96), and the mean BMI was 22.88 kg/m^2^ (*SD* = 6.54) (Table 1). The mean MPAI total score was 44.97 (*SD* = 11.88), the mean UCLA total score was 41.55 (*SD* = 9.75), and the mean SWLS total score was 22.10 (*SD* = 6.66) (Table 2). Internal consistency was good for all three scales, with Cronbach's α values of 0.881 for the MPAI, 0.890 for the UCLA Loneliness Scale, and 0.900 for the SWLS (Table 3).

**Table 1:** Participant characteristics.

Characteristic	Category	Overall (*N* = 1,311)
Age, years		19.31 ± 0.96
BMI, kg/m^2^		22.88 ± 6.54
Gender	Male	457 (34.86%)
	Female	854 (65.14%)
Grade	Freshman	808 (61.63%)
	Sophomore	503 (38.37%)
Ethnicity	Han	1,168 (89.09%)
	Ethnic minority	143 (10.91%)
Household registration	Urban	974 (74.29%)
	Rural	337 (25.71%)
Only–child status	Only child	785 (59.88%)
	Not only child	526 (40.12%)
Monthly disposable living expenses, CNY	< 1,000	32 (2.44%)
	1,000–1,499	209 (15.94%)
	1,500–1,999	394 (30.05%)
	2,000–2,499	382 (29.14%)
	2,500–2,999	114 (8.70%)
	≥3,000	180 (13.73%)
Self–reported relationship status	Not dating or married	1,056 (80.55%)
	Dating but not married	234 (17.85%)
	Married	3 (0.23%)
	Other	18 (1.37%)
Alcohol use	Frequent	23 (1.75%)
	Occasional	619 (47.22%)
	Never	669 (51.03%)
Tobacco use	Frequent	17 (1.30%)
	Occasional	55 (4.20%)
	Never	1,239 (94.51%)

Continuous variables are presented as M ± SD; categorical variables are presented as *n* (%). Monthly disposable living expenses were self-reported in six ordered categories denominated in Chinese yuan (CNY) and were entered as an ordinal covariate in the primary analysis. Relationship status was self-reported and was not independently verified. BMI, body mass index; CNY, Chinese yuan.

**Table 2:** Descriptive statistics for the primary study variables.

Variable	M	SD	Median	IQR	Minimum	Maximum
MPAI total score	44.97	11.88	45.00	16.00	17.00	78.00
UCLA total score	41.55	9.75	42.00	15.00	20.00	72.00
SWLS total score	22.10	6.66	22.00	9.00	5.00	35.00

All variables had *n* = 1,311. MPAI, Mobile Phone Addiction Index; UCLA, UCLA Loneliness Scale; SWLS, Satisfaction With Life Scale.

**Table 3:** Internal consistency reliability.

Scale	Items	Cronbach's α	McDonald's ω total
MPAI	17	0.881	0.880
UCLA Loneliness Scale	20	0.890	0.892
SWLS	5	0.900	0.909

Cronbach's α was the prespecified primary reliability coefficient; McDonald's ω total is provided as a supplementary estimate.

## Correlations among PMPU, loneliness, and life satisfaction

**Figure 1:**
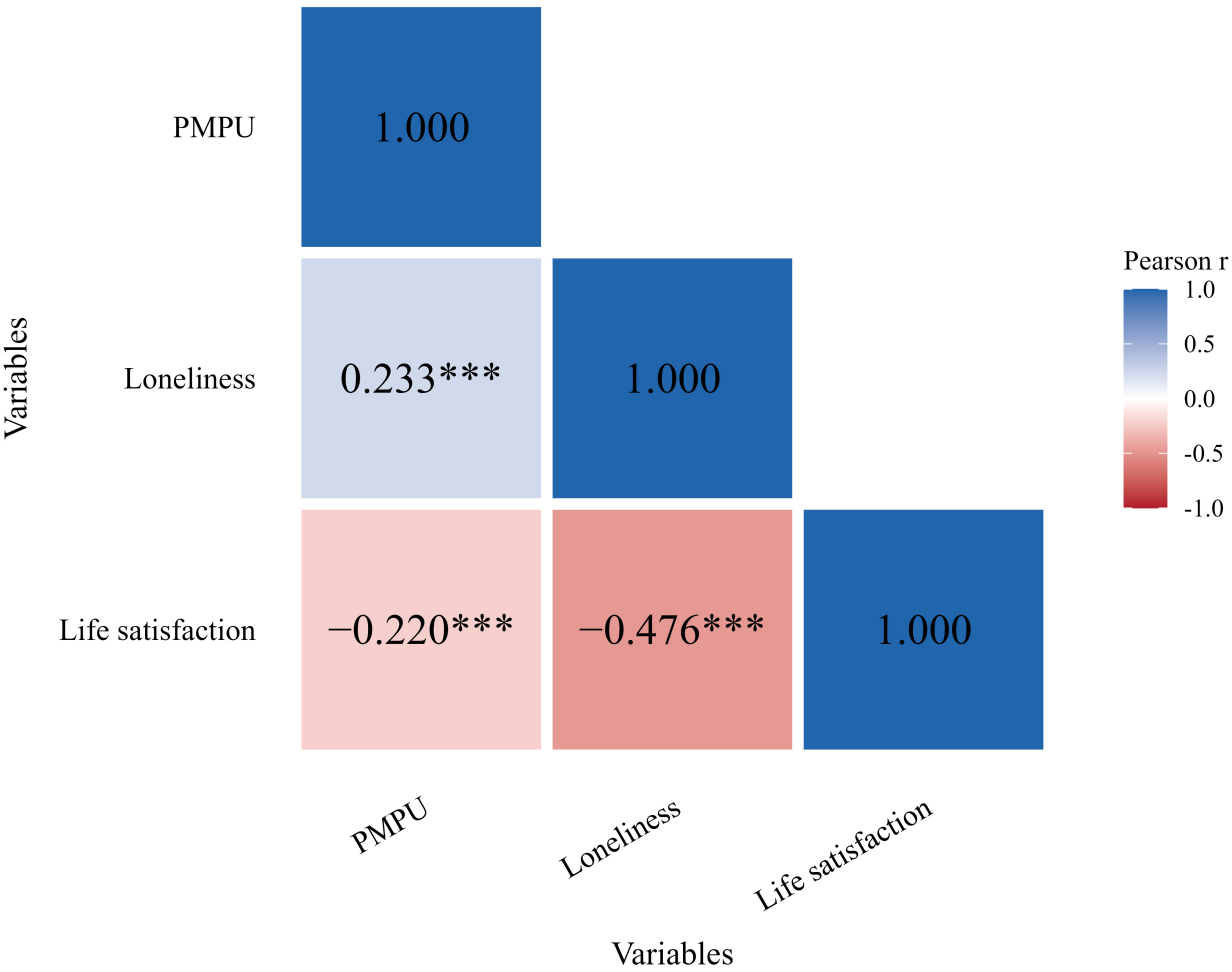
Correlation heatmap of problematic mobile phone use, loneliness, and life satisfaction. Cell colors indicate the direction and magnitude of Pearson correlation coefficients. Values in the cells are Pearson *r* coefficients. ***p < 0.001.

PMPU was positively correlated with loneliness [*r* = 0.233, 95% CI (0.181, 0.284), *p* < 0.001] and negatively correlated with life satisfaction [*r* = −0.220, 95% CI (−0.271, −0.168), *p* < 0.001]. Loneliness was also negatively correlated with life satisfaction [*r* = −0.476, 95% CI (−0.517, −0.433), *p* < 0.001]. Spearman correlation analyses yielded the same pattern of associations (Supplementary Table S1), and the Pearson correlations are visualized in Figure 1.

## Mediation analysis

In the adjusted OLS mediation model, PMPU was positively associated with loneliness (*a* = 0.205, *SE* = 0.022, *t* = 9.242, *p* < 0.001), and loneliness was negatively associated with life satisfaction after adjustment for PMPU and covariates (*b* = −0.281, *SE* = 0.017, *t* = −16.493, *p* < 0.001). The total association between PMPU and life satisfaction was−0.125 (*SE* = 0.015, *t* = −8.387, *p* < 0.001). After loneliness was included, the direct association was−0.068 (*SE* = 0.014, *t* = −4.829, *p* < 0.001). The corresponding covariate-adjusted regression paths are presented in Table 4.

**Table 4:** Covariate–adjusted OLS mediation paths.

Path	Association	B	SE	*t*	*p*	95% CI	*n*	Model *R*^2^
a	PMPU → loneliness	0.205	0.022	9.24	< 0.001	[0.161, 0.248]	1,311	0.111
b	Loneliness → life satisfaction, adjusted for PMPU	−0.281	0.017	−16.49	< 0.001	[−0.315, −0.248]	1,311	0.284
c	PMPU → life satisfaction, total association	−0.125	0.015	−8.39	< 0.001	[−0.155, −0.096]	1,311	0.133
c′	PMPU → life satisfaction, direct association	−0.068	0.014	−4.83	< 0.001	[−0.095, −0.040]	1,311	0.284

Coefficients are unstandardized. Models adjusted for gender, age, grade, ethnicity, BMI, household registration, only–child status, monthly disposable living expenses, relationship status, alcohol use, and tobacco use. All component equations used the same analytic sample.

**Figure 2:**
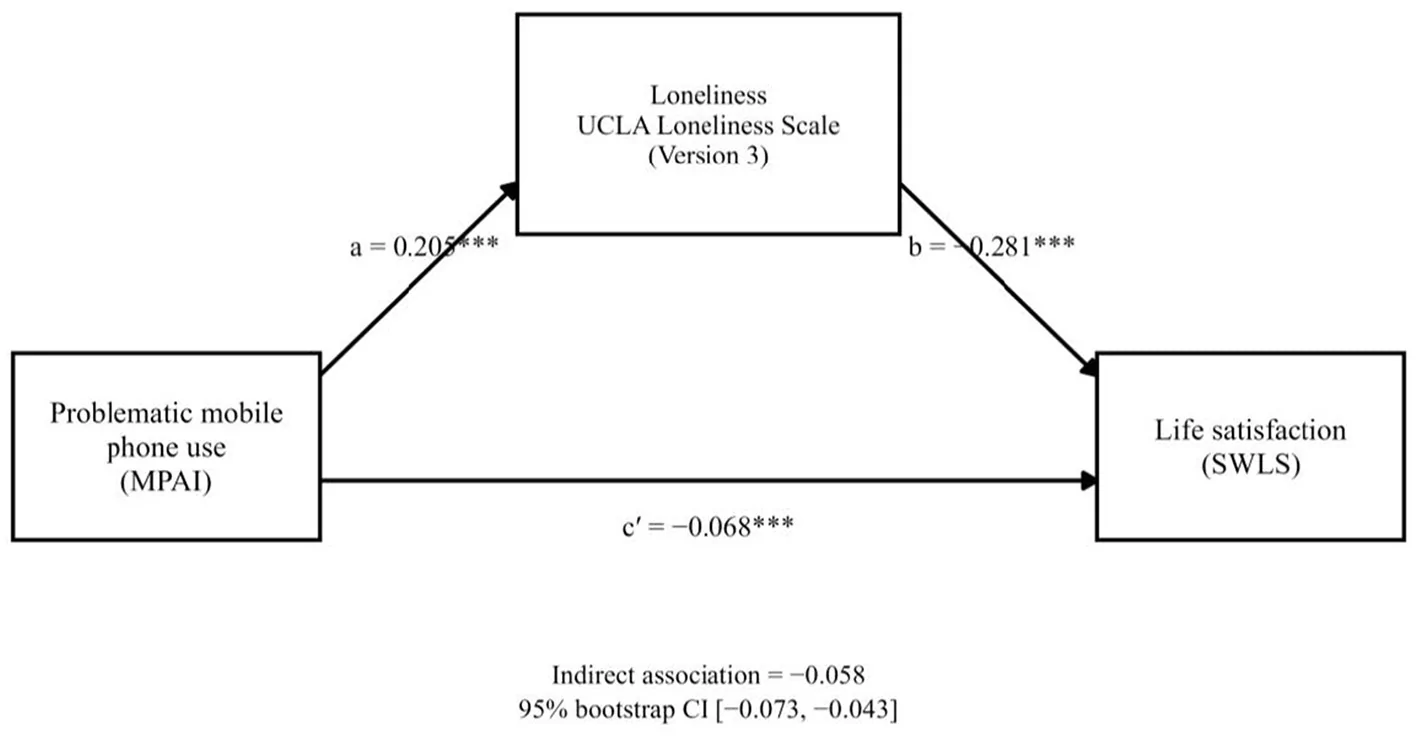
Covariate-adjusted observed-variable mediation model. Values are unstandardized regression coefficients. The indirect association was estimated with 5,000 percentile bootstrap samples. ***p < 0.001.

The bootstrap indirect association through loneliness was−0.058 [bootstrap SE = 0.007, 95% bootstrap CI (−0.073, −0.043)], accounting statistically for 45.9% of the total association (Table 5, Figure 2).

**Table 5:** Total, direct, and indirect associations.

Association	Estimate	SE/bootstrap SE	95% CI	Proportion mediated
Total association	−0.125	0.015	[−0.155, −0.096]	—
Direct association	−0.068	0.014	[−0.095, −0.040]	—
Indirect association	−0.058	0.007	[−0.073, −0.043]	45.9%

Estimates are unstandardized. The confidence interval for the indirect association was based on 5,000 percentile bootstrap samples. Cross–sectional indirect associations do not establish temporal ordering or causality. Proportion mediated was calculated from unrounded estimates; displayed coefficients may not sum exactly because of rounding.

## Sensitivity and SEM robustness analyses

The institution fixed-effects model yielded an indirect association of−0.059 [95% bootstrap CI (−0.074, −0.045)], and dummy coding of monthly expenses yielded−0.057 [95% bootstrap CI (−0.072, −0.043)]. The alternative directional model, with loneliness as the predictor and PMPU as the mediator, also produced a non-zero indirect association of−0.021 [95% bootstrap CI (−0.031, −0.011)], reinforcing the need for caution about directionality. The binary-screening and BMI-parsing sensitivity analyses were also substantively consistent with the primary model (Supplementary Table S2). The Breusch-Pagan test was non-significant (*p* = 0.270), the maximum adjusted generalized variance inflation factor was 1.279, and HC3 robust inference did not change the conclusions (Supplementary Table S5).

Women constituted 65.14% of the sample. The PMPU-by-gender interaction in the loneliness model was statistically significant [B = −0.096, SE = 0.046, 95% CI (−0.187, −0.006), *p* = 0.038]. The estimated indirect associations were −0.074 among men and −0.047 among women. However, a stable formal bootstrap estimate of the between-gender difference was not obtained; these exploratory results were therefore not interpreted as evidence of moderated mediation (Supplementary Table S6).

The three-factor CFA showed good fit, χ^2^ (62) = 259.54, CFI = 0.981, TLI = 0.976, RMSEA = 0.049 [90% CI (0.043, 0.056)], and SRMR = 0.033, and fit substantially better than the single-factor model, Δχ^2^ (3) = 3,949.29, *p* < 0.001. Adding a constrained common latent factor did not meaningfully improve fit relative to the three-factor model, Δχ^2^ (1) = 0.21, *p* = 0.643 (Supplementary Table S3, Panel A). In the unadjusted latent mediation model, the standardized indirect association was −0.141 [95% percentile bootstrap CI (−0.173, −0.108)]. The covariate-adjusted latent model also showed good fit, χ^2^ (227) = 575.65, CFI = 0.967, TLI = 0.960, RMSEA = 0.034 [90% CI (0.031, 0.038)], and SRMR = 0.030 (Supplementary Table S3, Panel B). Its standardized indirect association was −0.137 [95% percentile bootstrap CI (−0.173, −0.105)] (Supplementary Table S4, Supplementary Figure S1). All 1,000 requested bootstrap resamples were successful for the unadjusted model. For the adjusted model, 468 of 500 requested resamples were successful, whereas 32 failed or did not converge (Supplementary Table S4). The latent SEM findings were therefore interpreted as supplementary robustness evidence rather than evidence of temporal or causal mediation.

## Discussion

Our results showed a consistent pattern. Students with higher MPAI scores reported greater loneliness and lower life satisfaction, while loneliness was strongly associated with lower life satisfaction. In the adjusted model, loneliness accounted statistically for 45.9% of the total association between PMPU and life satisfaction. These findings are broadly consistent with previous research linking problematic digital use, loneliness, and subjective wellbeing in university students (30, 34–36, 38, 41–50).

The negative association between PMPU and life satisfaction should be understood in light of what the MPAI measures. During the lockdown, students depended on mobile phones for classes, communication, entertainment, and access to information, so frequent use alone was not necessarily problematic. The MPAI instead captures loss of control, distress when the phone is unavailable, use for withdrawal or escape, and interference with study or other responsibilities. Our findings therefore concern poorly regulated or disruptive use rather than ordinary or purposeful mobile phone use, which is consistent with previous studies linking problematic smartphone use to lower wellbeing (34, 35, 43–46).

Loneliness was closely related to the association observed between PMPU and life satisfaction. During the lockdown, reduced campus participation and face-to-face contact may have made social disconnection particularly relevant to students' life satisfaction. However, our data cannot determine the direction of this relationship. Compensatory Internet Use Theory suggests that people may turn to digital media for contact or relief when their offline needs are unmet (29), and the alternative-direction model also yielded a non-zero indirect association. The findings therefore leave several explanations open: PMPU may precede loneliness, loneliness may precede PMPU, or both may reflect shared stressors.

The association between digital-technology use and wellbeing remains contested. Twenge and Campbell (62) reported poorer psychological wellbeing among adolescents with heavier screen use, whereas Orben and Przybylski (63) found that the average associations were small and sensitive to analytic choices. These apparently different findings may reflect variation in how digital use is defined and measured, including its intensity, timing, purpose, and social context. Our study focuses specifically on problematic mobile phone use during the lockdown rather than screen exposure in general. The findings should therefore not be taken to mean that all mobile phone use is harmful.

How students used their phones may matter as much as the amount of time they spent on them. Different activities, purposes, and social contexts may have different associations with loneliness and life satisfaction (6, 24, 45, 46, 64). Because the present study assessed overall PMPU rather than specific patterns of phone use, we could not determine whether particular activities were more strongly related to either outcome. Future research could combine validated self-report measures with app-use records, diaries, or ecological momentary assessment to examine the content, timing, and purpose of mobile phone use more closely.

The sensitivity analyses yielded estimates similar to those of the primary analysis, including models with university fixed effects and alternative coding of monthly living expenses. These checks reduce concern that the findings were driven by those modeling choices, but they do not account for all possible differences among the seven participating institutions, such as lockdown rules, campus living conditions, online teaching arrangements, or access to psychological support. The PMPU-by-gender interaction in the loneliness model was exploratory. Although the estimated indirect associations differed numerically between men and women, a stable bootstrap estimate of the between-gender difference could not be obtained. We therefore did not interpret these results as evidence of moderated mediation. Replication in samples with a more balanced gender distribution is needed, particularly given previous reports of gender differences in problematic internet use and loneliness (65).

Although the data were collected during the Shanghai lockdown, the findings may have broader public health relevance because loneliness and prolonged social disconnection remain concerns among young people across different societies. The WHO has identified loneliness and social isolation as global public health priorities (66), while the United Kingdom and Japan have developed national policy responses to address loneliness and social isolation (67, 68). These developments suggest that strengthening social connection should remain an important component of university mental health services beyond the pandemic. At the same time, the transferability of our findings across countries should be considered cautiously, because cultural norms, family structures, campus environments, and patterns of digital-media use may shape how loneliness and problematic mobile phone use are experienced. The temporary and externally imposed restrictions examined in this study should also be distinguished from hikikomori, which involves prolonged and impairing social withdrawal (69). Nevertheless, both contexts raise a broader question for youth mental health in the digital age: whether digital media primarily helps socially isolated individuals maintain connection or, in some circumstances, reinforces avoidance and withdrawal. Our findings may therefore inform broader discussions of loneliness, social isolation, and digital self-regulation, while remaining specific to Chinese university students surveyed under lockdown conditions.

From a public health perspective, university support should address both digital self-regulation and social connection. Possible approaches include peer-support programs, counseling, physical activity, student clubs, and opportunities for students to develop meaningful offline relationships. These initiatives should not frame all mobile phone use as harmful, particularly because digital communication may provide important academic and social support. Instead, greater attention may be needed when students report difficulty controlling their use, interference with sleep or study, repeated reliance on mobile phones to avoid distress, or persistent loneliness.

## Strengths and limitations

This study has several strengths. Data were collected during a clearly defined period of strict restrictions and included students from seven public undergraduate colleges in Shanghai. Scale scores were recalculated directly from the original item-level responses, which allowed us to verify scoring and data consistency. The primary findings were also examined using alternative model specifications, regression diagnostics, confirmatory factor analysis, and supplementary structural equation modeling.

This study also has several limitations. The cross-sectional design does not establish temporal ordering or causality, and convenience sampling of freshmen and sophomores from seven colleges in Shanghai limits the generalizability of the findings. All variables were self-reported, leaving the results vulnerable to reporting bias and common-method variance. We also did not assess several potentially relevant factors, including academic stress, sleep quality, social support, self-control, depression, and anxiety. Relationship status was measured using a single item and did not capture relationship quality, duration, cohabitation, or partner support.

Women were overrepresented in the sample, and the exploratory gender findings require replication. Some MPAI items refer to mobile phone use when feeling isolated, lonely, or low in mood, which may partly overlap with the loneliness construct. In addition, we did not distinguish specific phone activities, purposes, or contexts of use. Because the survey was conducted during the 2022 Shanghai lockdown, the findings may not generalize to other periods or populations. Longitudinal and cross-lagged designs, ecological momentary assessment, and intervention studies are needed to clarify directionality and examine how social context shapes these associations.

## Conclusion

Among college students surveyed during Shanghai's 2022 lockdown, higher PMPU was associated with lower life satisfaction, and loneliness statistically accounted for part of this association. The alternative-direction analysis indicated that the ordering of PMPU and loneliness remains uncertain. Longitudinal and intervention research is needed to clarify directionality and identify approaches that support both social connection and healthier digital habits.

## Data Availability

The raw data supporting the conclusions of this article will be made available by the authors, without undue reservation.
