# Supplementary material for: Problematic mobile phone use, loneliness, and life satisfaction among college students during the COVID-19 lockdown in Shanghai: a cross-sectional mediation analysis
Source: Front Public Health. 2026 Aug 18;14:1925515. doi: 10.3389/fpubh.2026.1925515 (PMC13529553; doi:10.3389/fpubh.2026.1925515)
Supplement: Supplementary file 1 [file Supplementary_File_1.docx]

**Supplementary Materials**

*Problematic Mobile Phone Use, Loneliness, and Life Satisfaction among College Students during the COVID-19 Lockdown in Shanghai*

1. Supplementary Tables and Figure

Supplementary Table S1. Correlations among the primary variables

| **Variable 1** | **Variable 2** | **n** | **Pearson r** | **95% CI** | **p** | **Spearman’s ρ** | **p** |
| --- | --- | --- | --- | --- | --- | --- | --- |
| PMPU (MPAI) | Loneliness (UCLA) | 1,311 | 0.233 | [0.181, 0.284] | < 0.001 | 0.223 | < 0.001 |
| PMPU (MPAI) | Life satisfaction (SWLS) | 1,311 | −0.220 | [−0.271, −0.168] | < 0.001 | −0.201 | < 0.001 |
| Loneliness (UCLA) | Life satisfaction (SWLS) | 1,311 | −0.476 | [−0.517, −0.433] | < 0.001 | −0.467 | < 0.001 |

*Note.* Pearson correlations were primary; Spearman correlations were sensitivity checks. All tests were two-sided.

Supplementary Table S2. Sensitivity analyses of the indirect association

| **Model** | **Indirect association** | **Bootstrap SE** | **95% bootstrap CI** | **Proportion mediated** |
| --- | --- | --- | --- | --- |
| Primary model | −0.058 | 0.007 | [−0.073, −0.043] | 45.9% |
| Binary PMPU screening classification | −0.924 | 0.168 | [−1.261, −0.598] | 56.5% |
| Alternative-direction model | −0.021 | 0.005 | [−0.031, −0.011] | 6.8% |
| Institution fixed-effects model | −0.059 | 0.008 | [−0.074, −0.045] | 46.2% |
| Monthly expenses dummy-coded model | −0.057 | 0.007 | [−0.072, −0.043] | 45.9% |
| BMI parsing sensitivity model | −0.058 | 0.008 | [−0.072, −0.044] | 45.9% |

*Note.* The alternative-direction model is a sensitivity analysis and does not establish temporal ordering or causality. Proportions mediated are interpreted within each model and should not be compared directly across differently scaled or differently directed models.

Supplementary Table S3. CFA and latent SEM model fit

| **Panel / model** | **n** | **χ²** | **df** | **χ²/df** | **CFI** | **TLI** | **RMSEA** | **RMSEA 90% CI** | **SRMR** | **AIC** | **BIC** |
| --- | --- | --- | --- | --- | --- | --- | --- | --- | --- | --- | --- |
| **Panel A. CFA measurement models** |  |  |  |  |  |  |  |  |  |  |  |
| Single-factor CFA | 1,311 | 4208.831 | 65 | 64.751 | 0.594 | 0.513 | 0.221 | [0.215, 0.226] | 0.167 | 41231.063 | 41365.706 |
| Three-factor CFA | 1,311 | 259.538 | 62 | 4.186 | 0.981 | 0.976 | 0.049 | [0.043, 0.056] | 0.033 | 37287.771 | 37437.948 |
| Constrained common latent factor CFA | 1,311 | 259.324 | 61 | 4.251 | 0.981 | 0.975 | 0.050 | [0.044, 0.056] | 0.033 | 37289.556 | 37444.913 |
| **Panel B. Latent mediation models** |  |  |  |  |  |  |  |  |  |  |  |
| SEM-1 unadjusted latent mediation | 1,311 | 259.538 | 62 | 4.186 | 0.981 | 0.976 | 0.049 | [0.043, 0.056] | 0.033 | 37287.771 | 37437.948 |
| SEM-2 covariate-adjusted latent mediation | 1,311 | 575.655 | 227 | 2.536 | 0.967 | 0.960 | 0.034 | [0.031, 0.038] | 0.030 | 53088.110 | 54015.070 |

*Note.* CFA, confirmatory factor analysis; SEM, structural equation modeling; CFI, comparative fit index; TLI, Tucker–Lewis index; RMSEA, root mean square error of approximation; SRMR, standardized root mean square residual; AIC, Akaike information criterion; BIC, Bayesian information criterion. The three-factor CFA fit substantially better than the single-factor CFA, Δχ²(3) = 3949.29, p < 0.001. Adding a constrained common latent factor did not meaningfully improve fit relative to the three-factor CFA, Δχ²(1) = 0.21, p = 0.643.

Supplementary Table S4. Standardized latent SEM paths

| **Model** | **a** | **b** | **c′** | **Indirect association** | **Bootstrap SE** | **95% bootstrap CI** | **Total association** | **Proportion mediated** | **Successful bootstrap resamples** |
| --- | --- | --- | --- | --- | --- | --- | --- | --- | --- |
| SEM-1 unadjusted | 0.292 | −0.484 | −0.129 | −0.141 | 0.017 | [−0.173, −0.108] | −0.270 | 52.3% | 1,000/1,000 |
| SEM-2 covariate-adjusted | 0.307 | −0.447 | −0.132 | −0.137 | 0.016 | [−0.173, −0.105] | −0.269 | 50.9% | 468/500 |

*Note.* Values are standardized. Percentile bootstrap confidence intervals were obtained by recalculating the standardized path coefficients within each bootstrap resample and were based on successfully converged resamples. All 1,000 requested resamples were successful for the unadjusted model. For the adjusted model, 468 of 500 requested resamples were successful; 32 failed or did not converge, and none were classified as nonadmissible. These cross-sectional models do not establish temporal ordering or causal mediation.

Supplementary Table S5. Regression diagnostics and HC3 robust-inference checks

| **Panel A. Diagnostic** | **Statistic / maximum** | **df** | **p** | **Screening threshold** | **Flagged observations** | **Interpretation** |
| --- | --- | --- | --- | --- | --- | --- |
| Breusch–Pagan test | 20.089 | 17 | 0.270 | — | — | No evidence of heteroskedasticity |
| Maximum adjusted GVIF | 1.279 | — | — | — | — | No meaningful multicollinearity |
| Cook’s distance | 0.037 | — | — | > 4/n = 0.003 | 64 | Maximum was well below 1 |
| Leverage | 0.424 | — | — | > 2p/n = 0.027 | 79 | High-leverage cases were reviewed |
| Externally studentized residual | 3.435 | — | — | \|residual\| > 3 | 5 | Five observations exceeded \|3\| |

*Note.* GVIF, generalized variance inflation factor. Influence thresholds were used as screening flags; observations were not deleted solely because they crossed conservative cutoffs. For leverage, p = 18 model parameters, including the intercept.

Panel B. HC3 robust-inference checks

| **Path** | **B** | **HC3 SE** | **t** | **p** | **Conclusion** |
| --- | --- | --- | --- | --- | --- |
| a: PMPU → loneliness | 0.205 | 0.024 | 8.710 | < 0.001 | Inference unchanged |
| b: Loneliness → life satisfaction | −0.281 | 0.017 | −16.512 | < 0.001 | Inference unchanged |
| c′: Direct PMPU association | −0.068 | 0.015 | −4.538 | < 0.001 | Inference unchanged |
| c: Total PMPU association | −0.125 | 0.016 | −7.809 | < 0.001 | Inference unchanged |

Supplementary Table S6. Exploratory gender analyses

| **Analysis** | **Group / term** | **Estimate** | **SE / bootstrap SE** | **95% CI** | **p / bootstrap result** |
| --- | --- | --- | --- | --- | --- |
| Gender distribution | Male | 457 (34.86%) | — | — | — |
| Gender distribution | Female | 854 (65.14%) | — | — | — |
| Interaction in loneliness model | PMPU × female | −0.096 | 0.046 | [−0.187, −0.006] | 0.038 |
| Interaction in life-satisfaction model | PMPU × female | −0.012 | 0.030 | [−0.070, 0.046] | 0.680 |
| Interaction in life-satisfaction model | Loneliness × female | −0.024 | 0.035 | [−0.092, 0.044] | 0.488 |
| Stratified indirect association | Male | −0.074 | 0.013 | [−0.101, −0.049] | 5,000/5,000 |
| Stratified indirect association | Female | −0.047 | 0.009 | [−0.066, −0.030] | 5,000/5,000 |

*Note.* The formal bootstrap estimate of the difference between male and female conditional indirect associations was not stable. These analyses were exploratory and do not establish moderated mediation.

Supplementary Figure S1. Covariate-adjusted latent mediation model

*Note.* Values are standardized structural coefficients. The standardized indirect association was −0.137 (95% percentile bootstrap CI [−0.173, −0.105]). Model fit was χ²(227) = 575.65, CFI = 0.967, TLI = 0.960, RMSEA = 0.034 (90% CI [0.031, 0.038]), and SRMR = 0.030. Of the 500 requested bootstrap resamples, 468 were successful; 32 failed or did not converge. The cross-sectional model does not establish temporal ordering or causality.
